# A Case Report of Atypical Hemolytic Uremic Syndrome Presenting With Disseminated Intravascular Coagulation

**DOI:** 10.7759/cureus.87437

**Published:** 2025-07-07

**Authors:** Hannah M Brink, Taylor M Wallworth, Scott W Penney, Javier A Padial, Hannah L Gale

**Affiliations:** 1 Pediatrics, Brooke Army Medical Center, San Antonio, USA; 2 Emergency Medicine, David Grant United States Air Force (USAF) Medical Center, Fairfield, USA

**Keywords:** alternate pathway complement, atypical hemolytic uremic syndrome, disseminated intravascular coagulation (dic), eculizumab, influenza a infection

## Abstract

A previously healthy seven-year-old boy presented with clinical and laboratory findings consistent with atypical hemolytic uremic syndrome (aHUS) given thrombocytopenia, microangiopathic hemolytic anemia (MAHA), and acute kidney injury in the setting of influenza A. Notably, he also met diagnostic criteria for disseminated intravascular coagulation (DIC) at the time of presentation with clinical findings including prolonged prothrombin time (PT), markedly elevated D-dimer, and low fibrinogen. While aHUS and DIC share overlapping clinical features, they are traditionally regarded as distinct entities, with aHUS driven by complement dysregulation and DIC by the widespread activation of the coagulation cascade resulting in microvascular thrombosis, consumptive coagulopathy, and secondary fibrinolysis. The patient was treated with both supportive care and eculizumab, a terminal complement inhibitor, leading to rapid and sustained clinical and laboratory improvement without recurrence. This case highlights the diagnostic and therapeutic complexity of concurrent aHUS and DIC, lends clinical support to emerging hypotheses that uncontrolled complement activation may contribute to DIC, and underscores the value of early recognition and complement-directed therapy in atypical hemolytic uremic syndrome (HUS).

## Introduction

Hemolytic uremic syndrome (HUS) is a thrombotic microangiopathy characterized by a triad of intravascular hemolysis, thrombocytopenia, and acute renal failure. It is the leading cause of acute kidney injury in children [1]. While classification systems for HUS are continually evolving in the literature, HUS can be further subdivided into typical and atypical causes. Typical HUS is triggered by Shiga toxin-producing *Escherichia coli* (STEC) infection, which accounts for approximately 90% of HUS affecting children [2].

Atypical HUS (aHUS) presents identically to typical HUS but results from uninhibited complement activation, often with an underlying genetic cause [3]. Unlike typical HUS, aHUS is a rare disease, with recent literature reporting an approximate annual incidence of 0.25-2 in 1,000,000 per year [1]. It is proposed that aHUS is precipitated by dysfunction in molecules that regulate the alternative complement pathway, resulting in elevated levels of C3 convertase, the overproduction of C3b molecules and C5 convertase, and the subsequent uninhibited formation of the membrane attack complex (MAC) [1,4]. Of note, complement factor B is required to create C3 convertase, and C3 convertase formation is regulated by complement factors H and I [1]. Ultimately, the formation of the MAC on endothelial cells, red blood cells, and platelets is responsible for the thrombotic microangiopathy and renal and endothelial damage common to HUS [1,2]. Genetically predisposed individuals, while typically asymptomatic, become susceptible to clinically significant aHUS when faced with physiologic conditions that enhance underlying alternative pathway activation, such as infection, autoimmune conditions, pregnancy, or certain medications [3].

Clinically distinct from aHUS is disseminated intravascular coagulation (DIC), which shares many features of aHUS but is distinguished by the hallmark of secondary fibrinolysis due to the activation and consumption of platelets, resulting in prolonged coagulation, elevated fibrin degradation products, and decreased fibrinogen levels [5]. In comparison, aHUS presents with thrombosis in the form of platelet activation and no altered coagulation [5,6].

## Case presentation

A seven-year-old previously healthy boy presented with three days of cough, congestion, and intermittent fevers up to 102 degrees Fahrenheit per a home thermometer. The day prior to presentation, he had dark-colored urine and two episodes of non-bloody, non-bilious emesis without diarrhea. He also experienced four episodes of self-resolving epistaxis. At presentation, associated symptoms included body aches, chills, and a mild intermittent headache responsive to acetaminophen. He did not have diarrhea, abdominal pain, costovertebral angle tenderness, gum bleeding, or bruising. He had a recent exposure to influenza at school but otherwise no recent travel or infectious exposures. He was an otherwise healthy child. He was taking a multivitamin supplement daily but had no prescription medication. There was no family history of bleeding dyscrasias.

Vital signs were notable for a fever of 38 degrees Celsius but with otherwise normal heart rate, respiratory rate, and blood pressure. His physical examination was notable for scattered petechiae on the bilateral ear lobes and the dorsum of the left foot. He was alert and appropriately interactive without other examination abnormalities.

Initial laboratory workup was significant for severe thrombocytopenia (7 × 10^3^/μL). There was also evidence of nonimmune hemolytic anemia, given low hemoglobin (10.6 g/dL), decreased haptoglobin (11 mg/dL), negative direct Coombs test, and peripheral smear notable for marked schistocytes and large platelets. Influenza A was detected via polymerase chain reaction (PCR) nasopharyngeal swab, and group A streptococcus was identified via PCR pharyngeal swab. High-sensitivity C-reactive protein was elevated (32.1 mg/L), indicating ongoing inflammation. A urinalysis revealed a dark-brown sample with proteinuria (2+), hematuria (3+), and six red blood cells per high-powered field. Renal function studies showed evidence of uremia with acute kidney injury (blood urea nitrogen of 30 mg/dL and creatinine of 0.8 mg/dL). Further hematologic laboratory findings were consistent with DIC, given prolonged prothrombin time (PT) and activated partial thromboplastin time (19.2 and 51.2 seconds, respectively), markedly elevated D-dimer (>20 mcg/mL fibrinogen equivalent units {FEU}), low fibrinogen (153 mg/dL), and elevated lactate dehydrogenase (>2,500 U/L). Creatinine kinase was normal. A renal and bladder ultrasound was unremarkable, and a chest X-ray was normal.

The activation and dysregulation of the alternative complement pathway were evidenced by increased total complement CH50 relative to baseline, low complement C3, elevated terminal complement complex sC5b-9, and elevated complement factor B despite high levels of the regulating complement factor H (CFH) autoantibody. There were normal levels of complement factor I. A disintegrin and metalloproteinase with a thrombospondin type 1 motif, member 13 (ADAMTS13) was normal, which ruled out thrombotic thrombocytopenic purpura. Antinuclear antibody (ANA) was negative, making an autoimmune etiology for abnormal complement activation unlikely. A stool PCR was unable to be obtained, though the absence of diarrhea or abdominal pain greatly decreased the clinical suspicion for a gastrointestinal infection. Please see Table 1 for all laboratory values.

**Table 1:** Laboratory results of the patient ADAMTS13, a disintegrin and metalloproteinase with a thrombospondin type 1 motif, member 13; FEU, fibrinogen equivalent units

Laboratory Findings	Patient Value	Reference Range
Platelets	7 × 10^3^/μL	150-400 × 10^3^/μL
Hemoglobin	10.6 g/dL	11.5-15.5 g/dL
Haptoglobin	11 mg/dL	30-200 mg/dL
High-sensitivity C-reactive protein	32.1 mg/L	≤5.0 mg/L
Blood urea nitrogen	30 mg/dL	6-23 mg/dL
Creatinine	0.80 mg/dL	0.31-0.61 mg/dL
Prothrombin time	19.2 seconds	12.3-14.6 seconds
Activated partial thromboplastin time	51.2 seconds	24.2-33.2 seconds
D-dimer	>20 mcg/mL FEU	0-0.49 mcg/mL FEU
Fibrinogen	153 mg/dL	213-462 mg/dL
Lactate dehydrogenase	>2,500 U/L	225-600 U/L
Creatinine kinase	223 U/L	39-308 U/L
Total complement CH50	>60 U/mL	>39 U/mL
Complement C3	71 mg/dL	90-180 mg/dL
Terminal complement complex sC5b-9	303.9 ng/mL	72-244 ng/mL
Complement factor B	436 mcg/mL	128-279 mcg/mL
Complement factor H autoantibody	7.5%	0%-7.3%
Complement factor I	40 mcg/mL	29-59 mcg/mL
ADAMTS13	77.4%	>10%

After initial assessment, the clinical diagnosis of atypical HUS was made given evidence of microangiopathic hemolytic anemia (MAHA) with prominent schistocytes on peripheral smear, thrombocytopenia, and acute kidney injury in the absence of lower gastrointestinal symptoms that would be concerning for typical STEC HUS. The initial interventions for this patient included a platelet transfusion, intravenous fluids, and broad-spectrum antibiotics. After admission to the pediatric intensive care unit, the patient continued to have intermittent fevers but otherwise remained hemodynamically stable. Additional initial interventions included the administration of vitamin K for coagulation support and oseltamivir for influenza A.

**Figure 1:**
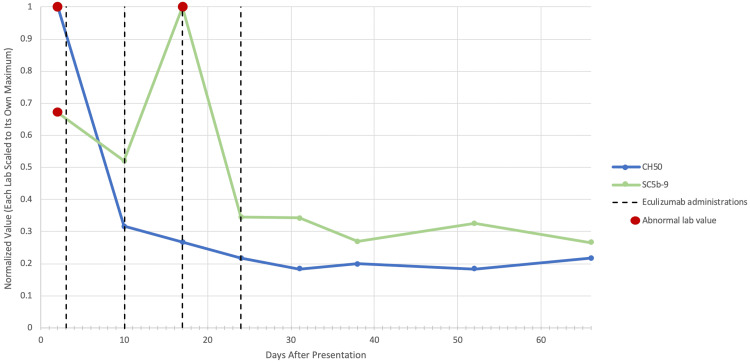
Representation of improvement and sustained normalization of total complement (CH50) and terminal complement complex levels (sC5b-9) with a full course of eculizumab Data are represented from the initial presentation, denoted as day 0. Laboratory values were normalized by dividing each value by the maximum observed value for that laboratory parameter, resulting in a rescaled range of 0-1. The red scatter plot markings indicate abnormally elevated complement activity. Eculizumab administrations are denoted by vertical dashed black lines

The DIC gradually trended toward correction with supportive care interventions. However, the platelets continued to downtrend with ongoing evidence of hemolysis and renal dysfunction, raising concern for ongoing aHUS. Given declining renal function and need for ongoing blood transfusion, the decision was made to initiate treatment with eculizumab 600 mg in weeks 1 and 2 and then 300 mg every two weeks for a total of four doses. He received the appropriate vaccinations to protect against encapsulated organisms prior to the administration of eculizumab. Within one day of receiving eculizumab, his markers of aHUS stabilized and began to normalize. He continued to receive eculizumab infusions as an outpatient with ongoing resolution of complement abnormalities (Figure 1). At the time of article submission, he remained off of therapy for 18 months without the recurrence of aHUS.

## Discussion

The presumed diagnosis for this patient is aHUS triggered by influenza A in the setting of an underlying abnormality in the complement system function. This case proved a diagnostic and therapeutic dilemma given that DIC is a distinct process that does not typically present alongside aHUS; though this phenomenon has been described, the physiologic mechanism has yet to be elucidated [7]. Kurosawa and Stearns-Kurosawa hypothesize that the overactivation of complement could induce aHUS, given that there has been similar uncontrolled activation of the complement system noted in DIC mouse models; thus, it would be reasonable to speculate that DIC could be a downstream effect of the overactive complement system in aHUS [6]. Expert opinion similarly theorizes that microvascular endothelial damage secondary to aHUS could precipitate a consumptive coagulopathy. Moreover, while influenza A has been described as an independent trigger of DIC, this is typically associated with severe cases of influenza and is reported sparingly in the literature as case reports [8,9]; the described patient was clinically stable with relatively mild influenza symptoms. Furthermore, a review of the current literature identified only a few published cases describing concurrent presentation of DIC with aHUS, all of which occurred in the setting of sepsis, a trigger that was notably absent in this patient [10-12]. All in all, the precise mechanism that precipitated simultaneous aHUS and DIC in this patient remains theory-based, though this case provides clinical support to the theory that the processes of aHUS and DIC may be linked.

Influenza is well-recognized as a trigger for aHUS [13]. Interestingly, however, in a review of 25 cases of influenza-associated thrombotic microangiopathy, a subset of eight patients underwent genetic testing as part of the workup (four with influenza A and four with influenza B), and all were found to have genetic abnormalities of the complement system [14]. The patient described in this case had high levels of CFH autoantibodies, which have been associated with the acquired dysfunction of CFH, a key negative regulator of the alternative complement pathway [15]. Genetic testing for this patient was ultimately obtained, and whole exome sequencing identified variants of unknown significance in cluster of differentiation 46 (CD46) c.565T>G and CFH c.2912C>T. While variants in CD46 and CFH have been reported in multiple individuals with aHUS, a subsequent genome-wide copy number variant analysis did not detect clinically significant copy number variants in this patient [15-17]. Therefore, there is no genetic explanation for his features at this time. However, this result may inform a future diagnosis given the rapidly progressing field of human genetics and frequent updates on variants of unknown significance. Furthermore, it has been shown that naturally occurring variations in CFH and CD46 are associated with a predisposition to aHUS in the setting of multiple independent risk factors prior to exposure to a specific trigger, and the majority of aHUS patients have not yet received a genetic diagnosis despite evidence to support its likelihood [13,18].

Atypical HUS can be a progressive disease associated with a poor prognosis. End-stage renal disease or death occurs in over 50% of patients within the first year after diagnosis due to the relapsing course of the disease after the initial triggering event [1,2,19]. Within the past decade, eculizumab has emerged as a valid treatment option for aHUS with improved outcomes associated with early administration [20]. Eculizumab is a humanized monoclonal antibody that functions as a C5 inhibitor to regulate complement activation [19]. The binding and inhibition of C5 halt the formation of the MAC, thus inactivating the alternative complement system and preventing further renal and endothelial damage [9]. A recent systematic review concluded that treatment with eculizumab may reduce dialysis rates and lead to improved renal function in individuals diagnosed with aHUS [19].

## Conclusions

This case illustrates the diagnostic and therapeutic challenges of concurrent aHUS and DIC, provides clinical support to emerging theories linking uncontrolled complement activation in aHUS to DIC, and emphasizes the importance of early recognition and complement-targeted therapy such as eculizumab to treat aHUS. Highlighted are the challenges associated with establishing an underlying genetic cause for predisposition to aHUS despite the likelihood of its presence. At the time of submission, the patient presented here has experienced 18 months of sustained resolution of aHUS without recurrence after treatment with eculizumab. Thus, this case supports existing evidence that the early administration of eculizumab can improve the long-term prognosis of aHUS, suggesting that patients with a high clinical suspicion for aHUS should ideally receive care at a facility capable of administering complement-targeted therapy. The findings of this case assist in expanding our understanding of complement-mediated aHUS, a rare and emerging disease.
